# Breastfeeding on ART and a closer look at drug transfer to HIV-exposed uninfected infants

**DOI:** 10.1038/s41598-025-20259-4

**Published:** 2025-09-18

**Authors:** Benedikt D. Spielberger, Alicia Zink, Jan C. Rohr, Susanne Usadel, Hartwig Klinker, Mirjam Freudenhammer, Roland Elling, Siegbert Rieg, Philipp Henneke, Markus Hufnagel, Matthias C. Müller

**Affiliations:** 1 Division of Pediatric Infectious Diseases and Rheumatology, Department of Pediatrics and Adolescent Medicine, Faculty of Medicine, University Medical Center, University of Freiburg, Freiburg, Germany; 2 Center for Chronic Immune Deficiency, Faculty of Medicine, University Medical Center, University of Freiburg, Freiburg, Germany; 3 Department of Infection Medicine, Medical Service Centre Clotten, Freiburg, Germany; 4 Division of Infectious Diseases, Department of Internal Medicine II, University of Würzburg Medical Center, Würzburg, Germany; 5 Institute for Infection Prevention and Control, Faculty of Medicine, University Medical Center, University of Freiburg, Freiburg, Germany; 6 Department of Medicine II (Gastroenterology, Hepatology, Endocrinology and Infectious Diseases), Faculty of Medicine, University Medical Center, University of Freiburg, Freiburg, Germany; 7 Breisacher Strasse 62, 79106 Freiburg, Germany; 8 Universitätsklinikum Freiburg, Breisacher Str. 86, D - 79110 Freiburg, Germany

**Keywords:** Breastfeeding, Maternal ART, Neutropenia, HIV-exposed, HIV-exposed uninfected (HEU), HIV infections, Paediatric research, Paediatric research

## Abstract

Adherence to antiretroviral therapy (ART) and maintaining an undetectable maternal HIV viral load effectively prevent intrauterine and perinatal HIV transmission 1, 2. However, concerns remain regarding the safety of breastfeeding in infants exposed to maternal ART. This retrospective study analyzed eight breastfed HIV-exposed uninfected (HEU) infants who were followed from January 2015 to July 2022. Maternal ART regimens included abacavir/lamivudine (n = 2), tenofovir alafenamide/emtricitabine (n = 1) or tenofovirdisoproxil/emtricitabine (n = 5) in combinations with rilpivirine (n = 3) (RPV), dolutegravir (n = 2) (DTG), raltegravir (n = 1), darunavir/ritonavir (n = 1), or nevirapine (n = 1). Infants and mothers underwent sequential HIV-PCR testing, complete blood count, and ART drug monitoring in breast milk, infant serum, and maternal serum when available. The relative infant dose (RID) was calculated to assess drug exposure. Hematological parameters were compared with those of 62 HEU formula-fed infants from the same institution. No HIV transmission occurred. ART serum levels in breastfed infants varied significantly, with RPV and DTG levels above the adult target range in two cases. Notably, one infant had RPV levels exceeding the target RID by 633%, leading to cessation of breastfeeding. At four months, mean absolute neutrophil counts were lower in breastfed infants (1,130/µl) compared to formula-fed infants (2,000/µl), with the lowest individual absolute neutrophil counts in the formula-fed group. Despite ART levels above the adult target range in 3 infants, there was no consistent pattern between neutropenia and elevated ART levels. Our findings highlight the substantial variability in infant ART exposure during breastfeeding. Given this variability and the potential for drug accumulation in individual cases, maternal therapeutic drug monitoring should be considered to identify elevated levels early on. In such situations, infant monitoring tailored to the individual may also be warranted.

**Supplementary Information:**

The online version contains supplementary material available at 10.1038/s41598-025-20259-4.

## Introduction

In resource-rich settings with access to antiretroviral therapy (ART), strict adherence during pregnancy has resulted in a vertical transmission rate of less than 1% among mothers living with HIV (MLWH), provided their viral load is suppressed before birth^1^.

To reduce the risk of postpartum HIV infection through breastfeeding, recommendations in resource-rich settings with easy access to infant formula advise against initiating breastfeeding. In recent years, however, breastfeeding has been reported to be safe in single-center studies and case reports from Germany and Switzerland^2–4^. Based on the results of the IMPAACT PROMISE study, the German guideline for care of HIV-exposed uninfected (HEU) infants was changed, supporting breastfeeding when the mother’s adherence to ART and successful ART are well documented^5,6^. A recent European survey of health workers revealed that while more MLWH are choosing to breastfeed, most national guidelines oppose it, and only less than half even address the option at all^7^.

Though breastfeeding likely promotes important health advantages for both the newborn and the mother, and the associated risk for HIV transmission appears very low, it remains unclear whether the amounts of different antiretroviral drugs transmitted through breast milk can impact on the infant’s health. In a systematic review from 2015, Waitt and colleagues showed that only relatively low levels of non-nucleoside reverse-transcriptase inhibitors (NNRTIs), nucleoside reverse-transcriptase inhibitors (NRTIs) and protease inhibitors (PIs) were transferred to the infants’ plasma^8^. Newer data from the Swiss Mother and Child HIV Cohort Study (MOCHIV) showed a highly variable level of transferred ARTs in the plasma of breast-fed infants^2^.

Studies from high-income countries indicate that HEU infants may face an increased risk of infections unrelated to HIV. While breastfeeding is known to offer general protective benefits, it is still unclear how exposure to ART during breastfeeding affects the infants’ vulnerability to these other infections^9,10^.

Here, we present data on ART exposure in a small, single-centre cohort of breastfed HEU infants and its implications for therapeutic drug monitoring (TDM).

## Methods

HIV-exposed infants were followed at the Pediatric Infectious Diseases Outpatient Clinic at the Department of General Pediatrics according to the current German and European guidelines for HIV-exposed or infected children^6,11^. MLWH were followed at the department of Infection Medicine, Medical Service Centre Clotten, Freiburg. The following provides a detailed overview of maternal ART regimens: Three mothers (infants 1, 6, and 7) received tenofovir disoproxil/emtricitabine 245/200 mg (TDF/FTC), and rilpivirine 25 mg (RPV) once daily. Two mothers (infants 2 and 4) were treated with dolutegravir 50 mg (DTG), abacavir 600 mg (ABC), and lamivudine 300 mg (3TC) once daily. The mother of infant 3 received tenofovir alafenamide/emtricitabine 25/200 mg (TAF/FTC) once daily in combination with nevirapine 200 mg (NVP) twice daily. The regimen for the mother of infant 5 included TDF/FTC 245 mg/200 mg and darunavir 800 mg boostered with ritonavir 100 mg (DRV/r), all administered once daily. The mother of infant 8 was treated with raltegravir 400 mg (RAL) twice daily and TDF/FTC 245/200 mg once daily.

For all HIV-exposed children visits, clinical examinations and blood sampling took place at the age of one, four and 18 to 24 months. Additionally, breastfed infants were seen at 14 days of age and for bi-monthly exams until cessation of breastfeeding. Routine blood tests included blood cell counts with differentials, transaminases, bilirubin at the first presentation, HIV-PCR and at 18–24 months or after cessation of breastfeeding HIV serology was performed. Primary outcome was infant serum drug exposure; breast-milk concentrations served as supportive secondary data. Serum samples of mothers and infants were drawn during visits to our clinic. Neither the timing of maternal drug intake and breastfeeding, nor the timing of the blood samples, was standardized. Therefore, no pharmacokinetic calculations could be performed. When available, additional serum samples of infants or mothers and a breast milk sample were sent to the Drug monitoring laboratory, Division of Infectious Diseases, Department of Internal Medicine II, University of Würzburg Medical Center, Germany. Antiretroviral drug monitoring for rilpirivine (RPV), nevirapine (NVP), dolutegravir (DTG), raltegravir (RAL) and darunavir/ritonavir (DRV/r) was performed using a validated high-performance liquid chromatography coupled with ultraviolet detection (HPLC-UV) method modified from Charbe et al.^12^. NRTI concentrations were not measured, as their active forms are intracellular metabolites, which are not detectable with standard plasma-based assays. To assess the infant’s ART drug exposure upon breastfeeding, we calculated the relative infant dose (RID). The RID is a commonly used tool for assessing drug exposure and is calculated by dividing the infant’s ART level by the mother’s ART level, expressed as a decimal or percentage^13,14^. All information for this study was derived from routine clinical exams and was, when available, retrospectively extracted from the patients’ medical records. As a control group, HEU infants who were not breastfed during the time of January 2015 to June 2022 were included in the analysis. For analysis, the HEU children were divided in 2 groups: HEU breastfed infants (*n* = 8) and HEU formula-fed infants (*n* = 62). The retrospective analysis was approved and informed consent was waived by the institutional review board of the University of Freiburg (22-1073-retro). All research was performed in accordance with relevant guidelines and regulations. Data collection and analysis were carried out in Microsoft Excel and R.

## Results

### Study population

In late 2018, the first MLWH decided to breastfeed her HEU infant. After one month, serum tests revealed RPV levels above the adult target range, which persisted at four months, prompting a recommendation to stop breastfeeding. Following this case, seven additional MLWH chose to breastfeed, and all eight mother-infant pairs were included in this analysis. Sixty-two HEU formula-fed infants seen at our clinic from January 2015 to July 2022 served as a control group for hematological parameters.

Infants 1 and 2 received zidovudine (ZDV) prophylaxis for four weeks. Infants 3–8 received two weeks of ZDV prophylaxis following the publication of new data indicating its safety and lower toxicity^15^. No HIV transmission was observed in any of the eight breastfed and sixty – two formula fed infants.

### Transmission of maternal ART to infant serum

ART drug monitoring was available at some point for all eight HEU breastfed infants. Complete data on ART levels in breast milk, maternal serum and infant serum were available on 13 occasions (Table 1). The RID is used for clinical risk assessment, with a target RID of below 10% indicating a low risk of adverse drug reactions in infants^16,17^.

#### Non-nucleoside reverse transcriptase inhibitors (NNRTIs)

RPV was measured in infants 1, 3, 6 and 7 and NVP in infant 7 (Table 1). In infant 1, maternal RPV levels were above the adult target range, corresponding to high serum levels at one and four months, with RIDs of 122% and 633%, respectively. Following cessation of breastfeeding, the infant’s serum levels fell below the detectable range by six months. In infants 6 and 7, the RPV serum levels were consistently below the range of detection, preventing accurate calculation of the RID, but allowing the assumption that the target RID was achieved. The RID for nevirapine in infant 3 was increased at 180% and 171% at 1 month and 4 months of age, respectively.

#### Integrase inhibitors (INI)

Infants 2 and 4 were exposed to DTG and infant 8 to RAL. In infant 2, serum DTG levels were above the target RID, with an RID of 240% at 4 months, despite maternal DTG levels being within the therapeutic range. In infant 4, DTG levels were consistently below the detectable range, resulting in a target RID of < 10%. In infant 8, the levels of RAL in the mother’s serum and breast milk were both in the adult target range. Correspondingly, the infant’s serum RAL levels remained below the detectable range, resulting in a target RID of < 10%.

##### Protease inhibitors (PI)

Infant 5 was exposed to DRV/r. The infant’s serum DRV/r levels were always below detectable levels, corresponding to an RID < 1%. Maternal DRV/r levels were in the adult target range on all occasions.

### Transmission of maternal ART to breast milk

Drug concentrations were measurable in seven out of 15 (47%) breast milk samples (Table 1). Five of the values fell within the adult trough range: DTG (648 and 680 ng/ml), RAL (293 and 168 ng/ml), and RPV (63 ng/ml). Two exceeded the upper trough limit: RAL at 14 days (1,757 ng/ml) and RPV at four months (4,300 ng/ml). The remaining eight samples were below the assay detection limits (50 ng/ml for NNRTI/INI and 250 ng/ml for PI).

### Alteration of hematological parameters in HEU infants

Complete and differential blood counts were analyzed in both HEU breastfed (*n* = 8) and formula-fed (*n* = 62) infants. Cases of cytopenia were observed in both groups. Given that all infants received ZDV prophylaxis, these findings may reflect known hematologic side effects of ZDV.

Additional ART exposure through breastfeeding could contribute to further cytotoxic effects. However, due to the small sample size and variability in ART regimens and serum levels, no consistent pattern between ART exposure and hematological changes can be concluded. Detailed neutrophil count comparisons are provided in Supplementary Fig. 1, and corresponding ART serum levels with neutrophil counts are shown in Supplementary Table 1. Comparisons of hemoglobin, thrombocyte, and leukocyte counts are presented in Supplementary Fig. 2.

## Discussion

Encouraging breastfeeding among MLWH in high-resource settings is a relatively recent development. Switzerland introduced this approach with its 2018 guideline revision, followed by Germany’s update in September 2020. The updated guidelines reduced barriers and provided clearer recommendations for both healthcare providers and patients.

In our cohort of eight breastfed and 62 formula-fed HEU infants, no cases of perinatal or postnatal HIV transmission occurred. This finding is consistent with other studies of breastfed HEU infants in resource-rich settings with good access to ART^2,18,19^.

Given the substantial evidence on the low risk of HIV transmission through breastmilk^20–22^, this information can be provided when counselling MLWH on their breastfeeding decision. However, when MLWH inquire about the impact of continued ART exposure by breastfeeding, data remain limited.

Pregnant women are strongly advised to take ART during pregnancy, making in utero exposure to these drugs inevitable. It is crucial to understand that during pregnancy, drug exposure (i.e., serum levels) in both the mother and the child is primarily influenced by maternal drug metabolism and excretion rates rather than those of the fetus. After birth, this dynamic changes, as the infant no longer relies on maternal metabolic capacity. This can result in cases where infant serum levels exceed those found in breast milk or maternal serum, as shown in mother-infant pairs 1 and 2 of this study.

ART levels can be elevated in breast milk, as observed for mother – infant pairs 1 and 8, leading to potentially higher uptake in infants compared to in utero. Drug metabolism in infants depends on glomerular filtration rates and metabolic enzyme activity, both of which can be lower than in adults^13,23^. Additionally, the duration of breastfeeding can vary significantly and may exceed the typical nine months of pregnancy. Consequently, the combination of drug accumulation in breast milk, reduced drug metabolism in infants, and extended breastfeeding may result in higher drug exposure than during pregnancy. This risk applies to a wide range of drugs, and drug-specific research in infants is limited due to ethical challenges.

The RID is a commonly used tool for assessing infant drug exposure during breastfeeding, with a threshold level below 10% generally assumed to confer little risk of side effects^16,17^. However, it should be emphasized that for most ART the evidence for this assumption is limited and that, consecutively, RID levels below or above 10% should not be automatically deemed safe or unsafe, respectively.

In the absence of robust and clear evidence, the effects of ART on the breastfed infant need to be assessed on a case-by-case basis to ensure that the benefits outweigh the risks, while avoiding unnecessary interruptions to breastfeeding.

Previous reports have also documented elevated levels of NVP in breastfed HEU infants^24,25^. Neutropenia is a rare side effect of NVP in children, and it can occur spontaneously in infants without identifiable risk^26^. Notably, infant 3 did not show neutropenia despite elevated NVP levels. RPV concentrations in Infant 1 were markedly elevated, with an RID of 633%, coinciding with maternal serum and breast milk levels well above the adult therapeutic range. Such supratherapeutic RPV concentrations could be due to unrecorded pharmacokinetic modifiers, most likely strong CYP3A4 inhibitors or agents that decrease gastric pH and enhance the absorption of RPV. However, our retrospective dataset lacks details of concomitant medications, preventing a definitive explanation. By contrast, Infant 7, whose mother’s RPV levels remained within the therapeutic window, exhibited minimal infant exposure (RID < 10%). These observations suggest a direct relationship between maternal serum RPV levels and infant exposure. Both RPV and NVP are highly lipophilic and readily transfer into breast milk, as we and other researchers have shown^2^. Their metabolism primarily depends on CYP3A4, an enzyme with significantly reduced activity in neonates and young infants^27^. While therapeutic maternal levels may be handled effectively by this limited enzymatic capacity, elevated maternal concentrations could exceed the infant’s metabolic clearance, thereby increasing the risk of drug accumulation.

The integrase inhibitors DTG and RAL are moderately lipophilic compounds capable of crossing biological membranes and entering breast milk. The elevated DTG levels in Infant 2 may reflect delayed metabolic clearance due to age-dependent activity of UGT1A1, a crucial enzyme responsible for integrase inhibitor glucuronidation. UGT1A1 reaches adult glucuronidation activity within the first few months after birth. However, this activity can vary between infants^28,29^, potentially accounting for the elevated DTG levels observed by us and others^30^. Although RAL is also metabolised via UGT1A1, no detectable infant serum levels were observed in Infant 8, despite elevated maternal and breast milk concentrations. This may reflect individual metabolic differences.

The low infant serum concentrations of DRV/r, as seen in Infant 5, are consistent with previous reports indicating poor transfer of protease inhibitors into breast milk^31,32^. Although DRV/r is lipophilic, its high plasma protein binding and possible efflux via breast cancer resistance protein (BCRP)^25^ - which transports substrates into breast milk^33^ - may limit its transfer.

Unlike HIV-infected adults, who benefit from ART through improved neutrophil counts and haematopoietic recovery, HEU infants have immune systems that are still maturing and may be altered by perinatal ART exposure. The long-term effects of such exposure during critical periods of immune development are not fully understood. Simultaneously, all infants in our study received the recommended ZDV prophylaxis, which is known to cause transient cytopenias. These hematologic changes may mask or confound potential toxicities from other ART agents. Integrase inhibitors, such as DTG and RAL, are considered low risk for direct cytotoxicity but may influence neutrophil function through interaction with RAG1, a gene expressed in neutrophils^34,35^. Protease inhibitors, such as DRV/r, exhibit anti-apoptotic as well as inhibitory effects on neutrophils, depending on intracellular accumulation and drug concentration^35^. In both cases interference with immunedevelopment is plausible, yet the clinical relevance of this remains uncertain.

Recent changes to the German guidelines have reduced the recommended duration of neonatal prophylaxis from four weeks to two, and the omission of ZDV prophylaxis is permitted in selected cases. This presents an opportunity to monitor hematological outcomes in the absence of ZDV and to better delineate the potential effects of ART exposure via breastfeeding in the future.

Our findings emphasize the potential importance of TDM in ART exposure during breastfeeding. Current guidelines in high-income countries like Germany or the United Kingdom state that routine HIV-PCR monitoring is mandatory for mother and child; however, TDM is not mentioned. This study does not establish causal links or toxicity thresholds. However, highly elevated infant ART levels suggest that maternal TDM may be justified in select cases - particularly for lipophilic drugs such as RPV and DTG.

Maternal TDM can be integrated into routine viral load assessments with minimal additional burden. Meanwhile, infant TDM poses more challenges due to limited blood volume and sampling feasibility. As significant accumulation was observed in only one infant, routine infant TDM cannot be widely recommended based on the limited available data. However, it should be considered when maternal serum levels are elevated or when clinical symptoms suggest toxicity. If clinically relevant infant drug levels are confirmed, temporary cessation of breastfeeding should be considered. Nevertheless, given the lack of evidence, we strongly recommend that such a decision is not based solely on ART levels, but also on the infant’s clinical appearance, as well as the mother’s needs and wishes.

Another interesting aspect for future research is whether drug levels in breast milk can be a reliable predictor of infant serum levels, thereby providing a non-invasive monitoring option in cases where no blood samples are taken or blood volume is insufficient. However, our study found that low breast milk ART levels did not consistently predict serum levels, as seen with DTG. Whether this holds true for other ARTs remains to be investigated.

It is important to note that the observed serum levels in this study were affected by variable timing of drug intake, breastfeeding and sample collection. Unlike in a controlled pharmacokinetic study, clinical practice rarely allows for the precise coordination of these factors. Unlike medication intake, breastfeeding follows the child’s needs rather than a fixed schedule, and blood draws depend on appointment availability. Consequently, the drug levels measured here reflect real-life variability. These limitations must be considered when interpreting individual serum concentrations. Nevertheless, when maternal TDM is performed, we strongly recommend documenting the timing of drug intake relative to sample collection to improve the reliability of result interpretation.

Further limitations include the reliance on single-point measurements and missing data presents challenges in interpreting ART exposure and its effects. In most cases, information on infant vaccination dates, birth height and weight percentiles, gestational age at birth, co-medications (other than vitamin D), and parental ethnicity was unavailable. Third, specialised assays to detect intracellular NRTIs were unavailable, so these data are absent. Further studies should address the transfer of NRTIs and the related risk of toxicity.

Despite these limitations, the strengths of the study include the examination of multiple ART regimens, offering a broad view of how different ARTs may impact breastfed infants. This diversity of ART exposure provides valuable insights into the pharmacokinetics and safety of breastfeeding while on ART for MLWH.

## Conclusions

Our findings emphasize the complexity of antiretroviral drug exposure during breastfeeding, as well as the significant variability in infant drug levels between individuals. Infants with ART levels above the 10% RID highlight the need for personalized monitoring. Due to current limitations in the available data, maternal TDM, particularly for drugs with high lipophilicity or long half-lives, may help to identify risk constellations at an early stage. Future research should focus on defining actionable exposure thresholds for infants, optimizing non-invasive monitoring tools and clarifying the long-term outcomes of ART exposure during breastfeeding.

**Table 1:** ART levels at 14 days, 1, 4 and 6 months after birth in maternal serum (MS), breast milk (BM) and infant serum (IS) in ng/ml for non-nucleoside reverse transcriptase inhibitors (NNRTI), protease inhibitors (PI) and integrase inhibitors (INI). Breastfeeding (BF) mode is categorized as exclusive (Excl), partial (Part), or stopped/switched to formula (Stop). Missing data are indicated by a single line. Target range for drug trough levels in adults and target RID (< 10%) are shown as well, with MS values above the target range for trough drug levels and IS values above the target RID highlighted in bold.

		14 days of age				1 month of age					
Infant	NNRTI	BF	MS	BM	IS	BF	MS	BM	IS	Adult trough level [ng/ml]	< 10% RID [ng/ml]
1	RPV	Excl	-	-	-	Excl	**540**	**-**	**660**	50–200	< 20
3	NVP	Excl	-	-	-	Excl	-	-	**1208**	1600–6700	< 670
6	RPV	Excl	-	-	-	Excl	70	< 50	< 50	50–200	< 20
7	RPV	Excl	-	-	-	Excl	174	< 50	< 50	50–200	< 20
Infant	INI	BF	MS	BM	IS	BF	MS	BM	IS		
2	DTG	Excl	-	-	-	Excl	3650	< 50	-	250–4000	< 400
4	DTG	Excl	-	-	-	Part	544	-	< 50	250–4000	< 400
8	RAL	Excl	< 75	1757	< 75	Excl	**872**	293	< 75	75–550 *	< 55
Infant	PI	BF	MS	BM	IS	BF	MS	BM	IS		
5	DRV	Excl	-	-	< 50	Excl	3800	< 250	< 50	550–3800*	< 380
		4 months of age				6 months of age					
Infant	NNRTI	BF	MS	BM	IS	BF	MS	BM	IS	Adult trough level [ng/ml]	< 10% RID [ng/ml]
1	RPV	Excl	**1650**	4300	**10,450**	Stop	-	-	< 50	50–200	< 20
3	NVP	Excl	-	-	**1146**	Excl	3928	-	649	1600–6700	< 670
6	RPV	Excl	76	< 50	< 50	Excl	-	< 50	-	50–200	< 20
7	RPV	Excl	**230**	63	< 50	-	-	-	-	50–200	< 20
Infant	INI	BF	MS	BM	IS	BF	MS	BM	IS		
2	DTG	Excl	2410	648	**5780**	Part	-	680	**4040**	250–4000	< 400
4	DTG	Part	-	-	< 50	Stop	-	-	< 50	250–4000	< 400
8	RAL	Excl	184	168	< 75	-	-	-	-	75–550*	< 55
Infant	PI	BF	MS	BM	IS	BF	MS	BM	IS		
5	DRV	Excl	**4130**	< 250	< 50	Part	3365	< 250	< 50	550–3800* 000	< 380

*Cmax values for darunavir up to 8000 ng/ml and for raltegravir up to 4000 ng/ml may occur.

## Supplementary Information

Below is the link to the electronic supplementary material.

Supplementary Material 1

## Data Availability

The datasets used and/or analysed during the current study are available from the corresponding author on reasonable request.
