# Supplementary material for: Breastfeeding on ART and a closer look at drug transfer to HIV-exposed uninfected infants
Source: Sci Rep. 2025 Sep 18;15:32612. doi: 10.1038/s41598-025-20259-4 (PMC12446422; doi:10.1038/s41598-025-20259-4)
Supplement: Supplementary file 1 — Supplementary Material 1 [file 41598_2025_20259_MOESM1_ESM.docx]

**Supplementary** **Figure 1:** Box plots comparing mean neutrophil counts of formula-fed (blue non-filled dots) and breastfed (black filled dots) infants at 1, 4, and 18 months. Each box represents the interquartile range (IQR), with the median indicated by the horizontal line within the box. Whiskers show the minimum and maximum values, excluding outliers, while individual data points are overlaid to display distribution, with greater variability at 4 and 18 months.

**
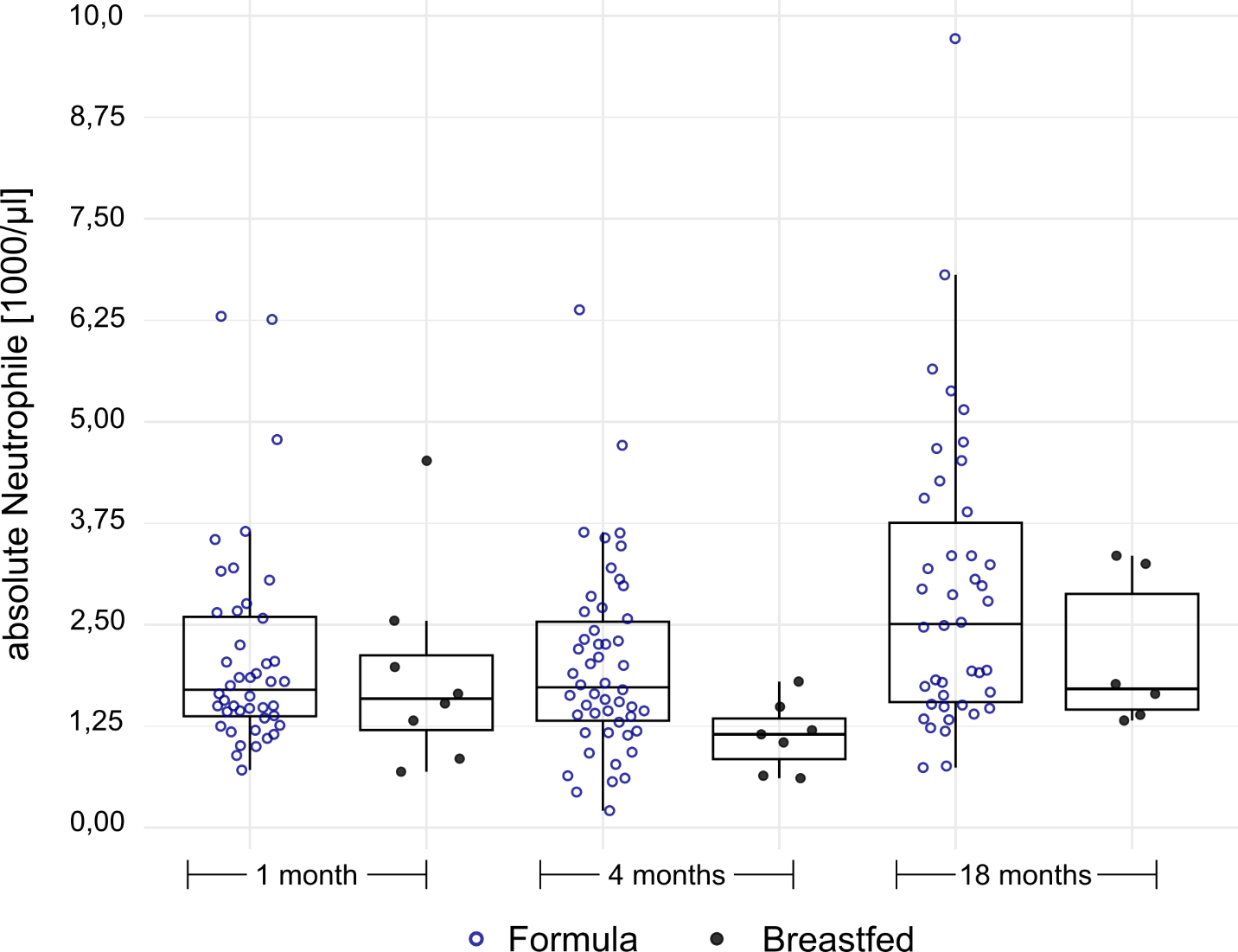
**

**Supplementary Table 1:** Comparison of infant serum (IS) ART levels in ng/ml with mean absolute neutrophil count (Neutro_abs) in 1000/µl for ART-exposed HEU breastfed infants, including nevirapine (NVP), darunavir/r (DRV), raltegravir (RAL), dolutegravir (DTG), and rilpivirine (RPV), at 14 days, 1, 2, 4, and 6 months, with Neutro_abs reassessed at 18 months. ART serum levels above a 10% RID of the adult target range and neutropenia <1,0 (1000/µl) are indicated in bold.

|  |  | **14 days** | | **1 month** | | **4 months** | | **6 months** | | **18 months** |
| --- | --- | --- | --- | --- | --- | --- | --- | --- | --- | --- |
| **Infant** | **ART** | **ART**  **_IS** | **Neutro**  **_abs** | **ART**  **_IS** | **Neutro**  **_abs** | **ART**  **_IS** | **Neutro**  **_abs** | **ART**  **_IS** | **Neutro**  **_abs** | **Neutro**  **_abs** |
|  |  | **ng**/**ml** | **1000**/ **µl** | **ng**/**ml** | **1000**/ **µl** | **ng**/**ml** | **1000**/ **µl** | **ng**/**ml** | **1000**/ **µl** | **1000**/ **µl** |
| 1 | RPV |  |  | **660** | 2,55 | **10450** | 1,05 | <50 | 1,39 | 1,39 |
| 2 | DTG |  |  |  | 1,98 | **5780** | **0,61** | **4040** | **0,97** | 3,25 |
| 3 | NVP |  |  | **1208** | 1,53 | **1146** | 1,20 | **649** | 3,33 | 1,32 |
| 4 | DTG |  | 3,53 | <50 | 4,52 | <50 | 1,49 | <50 | 6,44 |  |
| 5 | DRV | <50 | 1,16 | <50 | **0,69** | <50 | **0,64** | <250 | 1,15 |  |
| 6 | RPV |  | 2,25 | <50 | **0,85** | <50 | 1,80 |  | **0,94** | 1,77 |
| 7 | RPV |  | 2,27 | <50 | 1,65 | <50 | 1,15 |  | **0,74** | 1,65 |
| 8 | RAL | <75 | 2,74 | <75 | 1,32 | <75 |  |  |  | 3,35 |

**Supplementary** **Figure 2:** Box plots comparing hemoglobin (A), thrombocyte (B) and leukocyte and counts (C) of formula-fed (blue non-filled dots) and breastfed infants (black filled dots) at 1, 4, and 18 months. Each box represents the interquartile range (IQR), with the median indicated by the horizontal line within the box. Whiskers show the minimum and maximum values, excluding outliers, while individual data points are overlaid to display distribution.

**
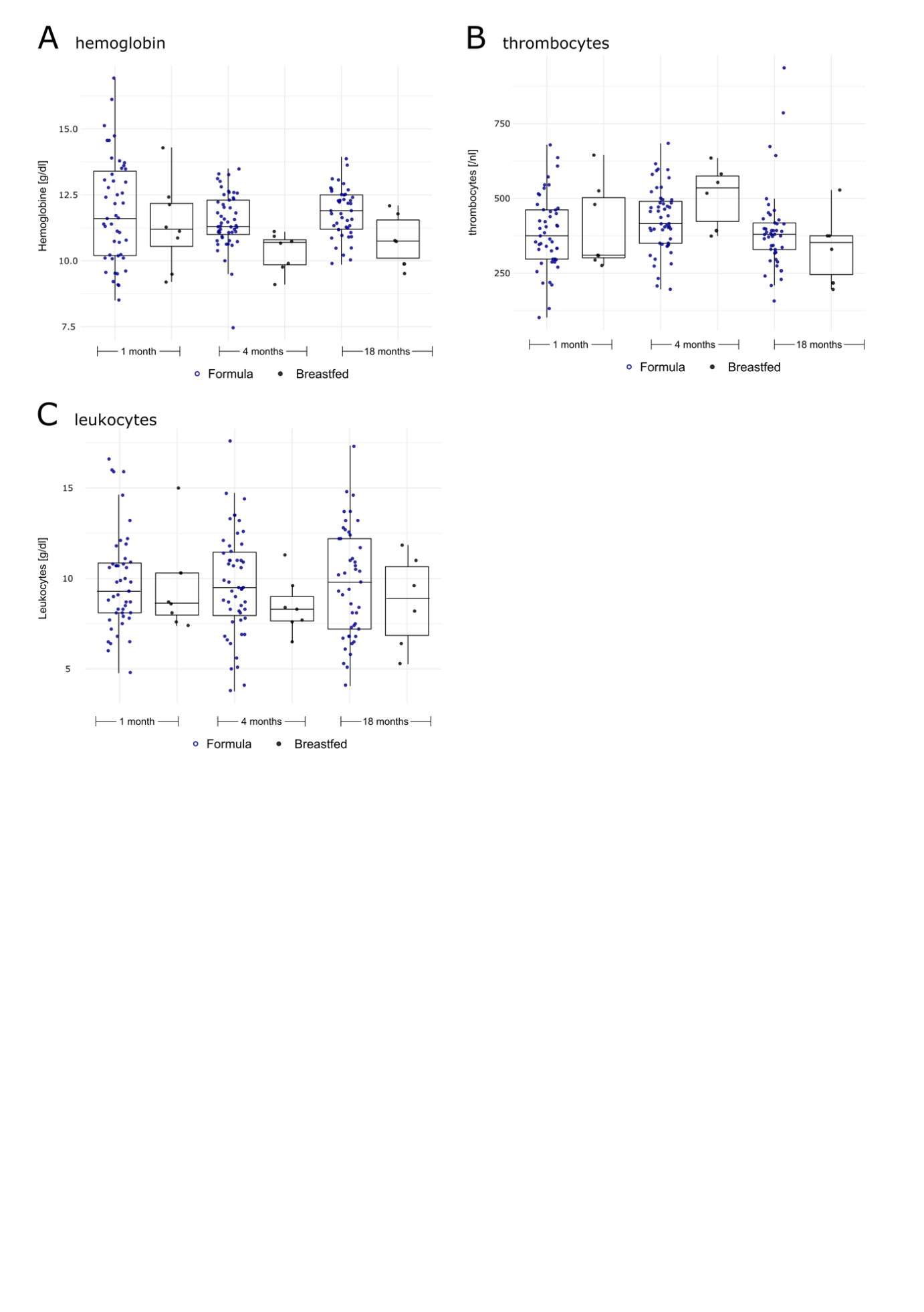
**
